# Correction: Selective area growth of GaN nanowires and nanofins by molecular beam epitaxy on heteroepitaxial diamond (001) substrates

**DOI:** 10.1039/d3na90031b

**Published:** 2023-03-20

**Authors:** Florian Pantle, Fabian Becker, Max Kraut, Simon Wörle, Theresa Hoffmann, Sabrina Artmeier, Martin Stutzmann

**Affiliations:** a Walter Schottky Institut and Physics Department, Technische Universität München Am Coulombwall 4 85748 Garching Germany florian.pantle@wsi.tum.de stutz@wsi.tum.de

## Abstract

Correction for ‘Selective area growth of GaN nanowires and nanofins by molecular beam epitaxy on heteroepitaxial diamond (001) substrates’ by Florian Pantle *et al.*, *Nanoscale Adv.*, 2021, **3**, 3835–3845, https://doi.org/10.1039/D1NA00221J.

The authors regret that in the “Morphology of GaN NSs on diamond” section on page 3837 the following text is incorrect (left column, lines 10–30):

“A similar morphology is observed for homoepitaxial GaN NWs grown on Ga-polar GaN templates. The almost hexagonal footprint with blunt edges of the NWs indicates that they are in a transition during the growth process from a dodecagonal shape with *m*- and *a*-plane side walls to their final, purely *m*-plane hexagonal shape with a pyramidal NW top. Compared to the NWs on the bare (111) and (001) diamond substrates, the NWs grown on an AlN buffer seem to be in a later state of this transition as they already show more defined *m*-plane side facets. This might be due to the more designated epitaxy of GaN on AlN as both have the wurtzite crystal structure. On the cubic diamond surfaces, the hexagonal formation of the NWs seems to be delayed or a complete formation might not even take place. This could also explain the different NW top facets as the tapered NW top evolves during the last phase of the hexagonal side facet formation due to minimization of surface energy. In contrast, NW tops are more flat in earlier facet formation states. A future nucleation study and an investigation of the influence of the growth duration on the NW morphology might help to better understand the growth kinetics.”

The correct text is the following:

“The cut lines of the top facets point toward the middle of the non-polar side facets like for *a*-plane NWs described in the article *J. Appl. Phys.*, 2022, **132**(18), 184304.^1^ Together with the blunt edges of their hexagonal cross section and the slightly inward bent side facets, we conclude that the NWs shown have *a*-plane side facets. The NWs on the bare (111) and (001) diamond substrates do not show such a pronounced hexagonal footprint. These deviations in the side facet formation for the different substrates might be due to differences in the local III–V ratio during MBE growth as described in the article *J. Appl. Phys.*, 2022, **132**(18), 184304.^1^ Probably, the different substrate temperatures during MBE growth resulted in variations of the main Ga diffusion length, which impacts the local Ga supply at the NSs.”

In addition, the following sentence in the “Photoluminescence properties” section on page 3841 (right column, lines 33–36) is incorrect:

“Thus, the 0.01 eV blue shift of the NFs can be explained by a larger extension of the stacking faults due to the 2D architecture of the fins compared to the more laterally confined NWs.”

The correct sentence is:

“The 0.01 eV blue shift of the NFs might be explained by a smaller lateral extension of the stacking faults compared to the ones in the NWs.”

The Royal Society of Chemistry apologises for these errors and any consequent inconvenience to authors and readers.

## Supplementary Material
